# Differential Flavonoids and Carotenoids Profiles in Grains of Six Poaceae Crops

**DOI:** 10.3390/foods11142068

**Published:** 2022-07-12

**Authors:** Jiaoyan Tang, Xukai Li, Yakun Zhang, Yulu Yang, Rong Sun, Yajun Li, Jianhua Gao, Yuanhuai Han

**Affiliations:** 1 Shanxi Key Laboratory of Minor Crop Germplasm Innovation and Molecular Breeding, College of Agriculture, Shanxi Agricultural University, Taigu 030801, China; tangjiaoyan_00@163.com (J.T.); zyakun0809@163.com (Y.Z.); yulu97_y@163.com (Y.Y.); lyajun0704@163.com (Y.L.); 2 Shanxi Key Laboratory of Minor Crop Germplasm Innovation and Molecular Breeding, College of Life Sciences, Shanxi Agricultural University, Taigu 030801, China; xukai_li@sxau.edu.cn (X.L.); sunrong19991024@163.com (R.S.); jhgao@sxau.edu.cn (J.G.)

**Keywords:** Poaceae, nutrition, flavonoids, carotenoids, metabolome

## Abstract

Poaceae practically dominate staple crops for humans. In addition to the issue of sustenance, there is a growing interest in the secondary metabolites of these staple crops and their functions on health. In this study, metabolomic variations were investigated among six important species of Poaceae with a total of 17 cultivars, including wheat, maize, rice, sorghum, foxtail millet, and broomcorn millet. A total of 201 flavonoid metabolites and 29 carotenoid metabolites were identified based on the UPLC-ESI-MS/MS system. Among them, 114, 128, 101, 179, 113, and 92 flavonoids and 12, 22, 17, 15, 21, and 18 carotenoids were found in wheat, maize, rice, sorghum, foxtail millet, and broomcorn millet, respectively. Only 46 flavonoids and 8 carotenoids were shared by the six crops. Crop-specific flavonoids and carotenoids were identified. Flavone, anthocyanins, flavanone and polyphenol were the major metabolite differences, which showed species specificity. The flavonoid content of the grains from 17J1344 (sorghum), QZH and NMB (foxtail millet) and carotenoids from Mo17 (maize) were higher than the other samples. This study provides a better knowledge of the differences in flavonoid and carotenoid metabolites among Poaceae crops, as well as provides a theoretical basis for the identification of functional metabolites in these grains.

## 1. Introduction

There is growing interest in improving the nutritional and medical value of foods. The color and health benefits of whole grains are linked to many secondary metabolites, such as carotenoids and flavonoids, etc., which exhibit potent bioactivities for either human health or plant development and defense [1]. 

Flavonoids are important pigments of many plants and contain several groups including flavones, flavanones, flavonols, flavanes, isoflavones, and anthocyanins [2,3]. These pigments show beneficial effects on humans or other organisms, such as anti-cancer activity, hypoglycemic effects and antioxidant activities, anti-bacterial activity, anti-inflammatory, and immune-modulatory activities [2]. For instance, apigenin, a flavone abundantly presented in celery, exhibited anti-inflammatory, anti-cancer, and anti-angiogenic properties [4,5,6]. Moreover, it also restored normal metabolic activity during inflammatory circumstances by modulating mitochondrial function [7]. Flavonoids were also thought to have possible nutritional benefits for feedstock, such as lowering lipid peroxidation in meats and rumen methanogenesis, as well as changing the structure of fatty acid in milk and meat [8]. In livestock production, flavonoids can also promote the production efficiency of ruminants, such as neonatal health, animal growth, dairy cows’ resilience to stress, the efficiency of rumen fermentation, and milk production [9]. 

Carotenoids are another important pigment molecule that play a variety of functions in plant growth and development. Carotenoids act as antioxidants and their ingestion has also been associated to lower risk of several chronic diseases. They are also important sources for health and pharmaceutical benefits and are industrially relevant biochemicals [10]. The evidence clearly showed a substantial inverse relationship between α-carotene, β-carotene, and lycopene intake with the incidence of prostate, stomach, and lung malignancies [11,12]. Ingestion of foods rich in carotenoids reduced the risk of type 2 diabetes, colorectal cancer, and obesity. Zeaxanthin and xanthophylls lutein were able to improve visual function of the age-related macular degeneration, and significantly protect against retinal vascular changes induced by hyperglycemia as well as the capillary cell degeneration [13,14]. β-carotene, retinol and lycopene have been found to prevent Alzheimer’s disease. In addition, lycopene also reduced the incidence of coronary heart disease and improved endothelial function [14]. 

The major Poaceae crops in the world are wheat (*Triticum aestivum*), maize (*Zea mays*), and rice (*Oryza sativa*). The constant improvement in the yield and quality of these crops is necessary to meet an ever-growing world population. Recently, the diversity of metabolites among staple food crops has been well documented. There are relatively similar flavonoid accumulation patterns among rice, wheat and maize, compared with other non-crop species such as Arabidopsis and tobacco [15]. The accumulation of specific flavonoids also affected the different nutritional value between fruits (including grape, mango and banana) and staple food crops (including rice, wheat, and maize) [16]. Carotenoids are important phytochemicals that provide nutritional benefits to cereal products, particularly the improvement of provitamin A carotenoids, which can alleviate vitamin A deficiency. For instance, rice has the lowest carotenoid content among all cereals, and the intake of polished rice-based foods with a non-diverse diets leads to vitamin A deficiency [17]. The carotenoid content in staple crops has species diversity. Maize contains a large amount of carotenoids and has extensive natural variation in genotypes [14]. Other crops, including sorghum (*Sorghum bicolor*), foxtail millet (*Setaria italica*), and broomcorn millet (*Panicum miliaceum*) are primarily consumed as the complement of the major crops especially in underdeveloped regions. Therefore, these crops are called minor crops. Since the minor crops are expected as the alternatives of the major crops and make up a fraction of the diet, their flavor and edible quality are of greater interest [18,19,20,21]. At present, the phenolic acids, flavonoids, and carotenoids have been identified in some minor crops which are especially important for those who are nutritionally deprived. The nutritional quality of foxtail millet is related to grain color, and the content of metabolites such as flavonoids and carotenoids affected the nutritional quality of foxtail millet [22]. The accumulation of flavonoids in broomcorn millet was variety-dependent. The composition and content of flavonoids may lead to different antioxidant activities of different varieties [23]. It is widely accepted that flavonoids and carotenoids are beneficial to human health, whereas the diversity of nutritional metabolites between staple food crops and minor crops is largely unknown.

In this study, we investigated the differences of the flavonoids and carotenoids in the grains of the six crops described above by using the UPLC-ESI-MS/MS. Crop-specific flavonoids and carotenoids were identified. Flavone, anthocyanins, flavanone, and polyphenol were the major metabolite differences, which showed species specificity. The study will shed light on the understanding of the characteristic functions of the minor crops and promote their popularization.

## 2. Materials and Methods

### 2.1. Plant Materials

The 17 cultivars of six Poaceae crops in this study were wheat-JM47 (Jinmai47, commercial variety), JM22 (Jimai22, medium gluten), SL02 (Shiluan02-1, strong gluten); maize-B73 (female line, reference genome available), Mo17 (male line, reference genome available); rice-NPB (japonica cultivar, reference genome available), 93-11 (indica cultivar); sorghum-BTx623 (reference genome available), 17J90 (edible), 17J1344 (brewing); foxtail millet-JG21 (Jingu21, cultivar of high-quality conventional, reference genome available), QZH (Qinzhouhuang, cultivar of typical representative), YG1 (Yugu1, cultivar, reference genome available), NMB (Niumaobai, landrace), DBQ (Daobaqi, landrace); broomcorn millet: LM4 (Longmi4, reference genome available), JS9 (Jinsu9, waxy grains cultivar) (Table 1). 

The dried grain samples used in the experiment were obtained from the Shanxi Agricultural University (foxtail millet), Shanxi Academy of Agricultural Sciences (wheat and sorghum), Huazhong Agricultural University (rice and maize) and Gansu Academy of Agricultural Sciences (broomcorn millet), which were harvested in 2018. 

The mature grains were used for metabolic profiling detections in the same year after harvested. For each sample, more than 3 g room temperature stored grains were randomly selected. 

### 2.2. Chemicals

The Key chemical reagents were chromatographic-grade. Gradient grade of aqueous methanol, acetonitrile, ethanol, and acetic acid were purchased from Merck (Darmstadt, Germany). MTBE and n-hexane were purchased from CNW (Shanghai, China). Acetone purchased from Sinopharm Chemical Reagent Co., Ltd. (Shanghai, China). Formic acid and lidocaine were purchased from Sigma-Aldrich (Shanghai, China), Butylated Hydroxytoluene were purchased from Aladdin (Shanghai, China). All reagents are stored in accordance with storage conditions and requirements. In addition, water was doubly deionized with a Milli-Q water purification system (Millipore, Bedford, MA, USA).

### 2.3. Sample Preparation and Extraction for Flavonoid metabolites 

The sample was freeze-dried, ground into powder (30 Hz, 1.5 min) using a grinder machine (MM400, Retsch), and stored at −80 °C until needed. Flavonoids were extracted refer to the method as previously described [24]. Briefly, 0.1000 g of grains flour was extracted with 1.0 mL 70% aqueous methanol (including 0.1 mg/L lidocaine) at 4 °C overnight. It was then centrifuged at 14,000 rpm for 5 min at 4 °C. The supernatant fractions were absorbed on a CNWBOND Carbon-GCB SPE Cartridge (ANPEL, Shanghai, China) and then filtrated with SCAA-104 (ANPEL, 0.22 µm pore size, Shanghai, China) before UPLC-ESI-MS/MS analysis. 

### 2.4. UPLC Conditions for Flavonoid Metabolomics Analysis

The flavonoid metabolomics of samples were analyzed as described previously [3], using a UPLC-ESI-MS/MS system (UPLC, Shim-pack UFLC SHIMADZU CBM30A system; MS, Applied Biosystems 6500 Q TRAP, Foster City, USA) equipped with a C18 chromatographic column (Waters ACQUITY UPLC HSS T3, 2.1 mm × 100 mm, 1.8 µm). Phase A of the mobile phase (0.04% acetic acid in water) and phase B of the mobile phase (0.04% acetic acid in acetonitrile) were used as solvent systems. The following was the gradient program (A:B): 95:5 (*v/v*) at 0 min, 5:95 (*v/v*) at 11.0 min, 5:95 (*v/v*) at 12.0 min, 95:5 (*v/v*) at 12.1 min, 95:5 (*v/v*) at 15.0 min, and 95:5 (*v/v*) at 15.0 min; flow rate 0.40 mL/min. The injection volume was 2.0 µL, and the column temperature was held at 40 °C. A Q TRAP-MS was used to analyze the effluent. 

### 2.5. ESI-Q TRAP-MS/MS System for Flavonoid Metabolomics Analysis

The mass spectrometry (MS) referred to the method of Chen et al. [25]. To detect metabolites, a triple quadrupole-linear ion trap mass spectrometer (Q TRAP, AB Sciex, Foster City, CA, USA) was used with an API 6500 Q TRAP LC/MS/MS System equipped with Linear ion trap (LIT) and triple quadrupole (QQQ) scans. Ion source gas I, gas II, and curtain gas pressures were set at 55.0, 60.0, and 25.0 psi, respectively. For instrument troubleshooting and mass calibration, 10.0 µmol/L and 100.0 µmol/L polypropylene glycol solutions were utilized in the QQQ and LIT modes. Multiple reaction monitoring (MRM) mode was used to execute QQQ scanning with the collision gas (nitrogen) set to 5.0 psi. The de-clustering potential (DP) and collision energy (CE) for each MRM transition were completed by further optimizing. The distinct set of MRM was detected according to the metabolites eluted in each time.

### 2.6. Qualitative and Quantitative Analysis of Flavonoids Content

The quantitative analysis of the metabolites followed the protocols of Fraga [26] and Wang [3]. Based on the Metware database, the primary and secondary spectral data from mass spectrometry were analyzed qualitatively. The quantitative measurement of metabolites was carried out using QQQ mass spectrometry in MRM mode. Then the chromatographic peaks of all the samples were integrated for quantitative analysis. 

### 2.7. Sample Preparation and Extraction for Carotenoid metabolites

The freeze-dried samples were ground into powder for 1.5 min at 30 Hz and stored at −80 °C until needed. Fifty milligrams of powder of each sample was extracted by 0.5 mL mixed solution of n-hexane: acetone: ethanol (1:1:1, *v/v*/*v*) for the quantification. As an internal standard, ten microliters (20 μg/mL) of internal standard mixed solution were added to the extract. At room temperature, the extract was vortexed for 20 min. After centrifuging at 12,000 rpm for 5 min at 4 °C, the supernatants were collected. The residue was re-extracted in the same condition. The supernatant of two extracts was combined and then evaporated to dryness, reconstituted in mixed solution of MeOH/MTBE (1:1, *v/v*). For LC-MS/MS analysis, the solution was filtered via a 0.22 μm membrane filter [27,28,29]. 

### 2.8. UPLC Conditions for Carotenoid metabolites 

The extracts were analyzed by UPLC-APCI-MS/MS system (UPLC, ExionLC™AD, https://sciex.com.cn (accessed on 4 July 2022); MS, Applied Biosystems 6500 Triple Quadrupole, https://sciex.com.cn/ (accessed on 4 July 2022)). The analytical conditions for LC were as follow: Column, YMC 3 μm, 100 mm × 2.0 mm C30; Solvent system, acetonitrile: methanol (3:1, *v/v*) with phase A (0.01% Butylated Hydroxytoluene and 0.1% formic acid), phase B (methyl tert-butyl ether with 0.01% Butylated Hydroxytoluene); Gradient program, 0–3 min started at 0% B, 3–5 min increased to 70% B, 5–9 min increased to 95% B, 10–11 min ramped back to 0% B; Flow rate, 0.8 mL/min; Temperature, 28 °C; Injection volume: 2 μL [29,30]. 

### 2.9. APCI-MS/MS Conditions for Carotenoid metabolites

On QTRAP^®^ 6500^+^ LC-MS/MS System (Shimadzu, Kyoto, Japan), equipped with an APCI Heated Nebulizer, operated in positive ion mode, and controlled by Analyst (1.6.3) software; linear ion trap (LIT) and triple quadrupole (QQQ) scans were acquired. The APCI source operation parameters were as follows: ion source, APCI+; source temperature at 350 °C; curtain gas (CUR) was set at 25.0 psi. Carotenoids were analyzed using scheduled MRM. According to the metabolites eluted during each phase, a specific set of MRM transitions was observed [29,31]. All metabolites detected were quantified using Multiquant 3.0.3 software. The software Analyst (1.6.3) was used to collect the data. Further optimization of mass spectrometer parameters such as the DP and CE for particular MRM transitions was performed. 

### 2.10. Quality Control Analysis of Samples

The samples were processed and tested in the same way. The stability of the instruments ensured the data’s reproducibility and reliability. Mixing equal amounts extractions with three groups was used to test the quality of different cultivars. Three repetitions for each sample. A quality control sample was inserted between 10 testing samples to ensure reproducibility. 

### 2.11. Statistical Analysis

Metabolites that differed significantly between groups were discovered by absolute Log2FC and *t*-test *p*-value. Metabolites identified in all samples were used for hierarchical clustering analysis (HCA), volcano plot, and PCA. Microsoft Office Excel 2019 and R (4.1.1, http://www.r-project.org (accessed on 10 October 2021)) were used to conduct the statistical analysis. The significance of differences was evaluated by the Duncan’s multiple range test. Significance was set at *p* < 0.05.

## 3. Results

### 3.1. Metabolic Profiling in Different Species

**Figure 1:**
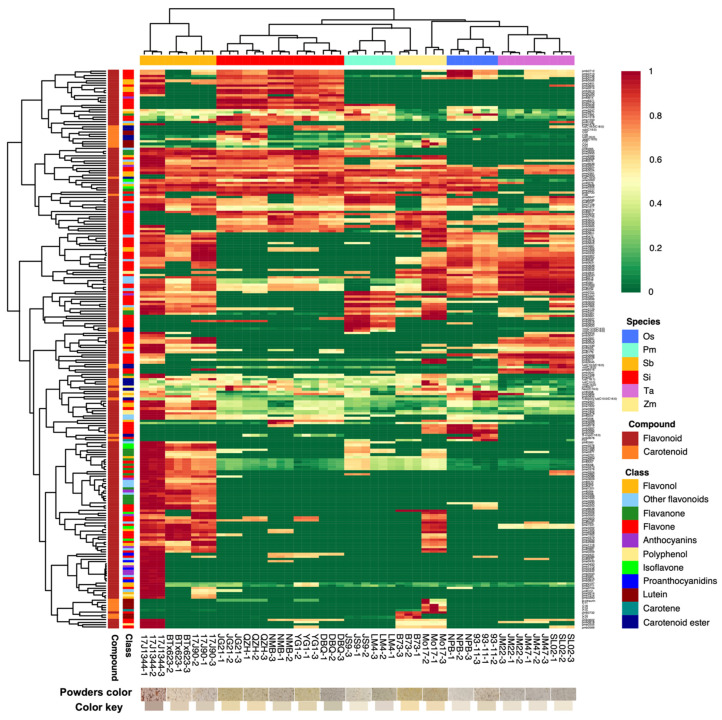
Clustering Heatmap of all flavonoids and carotenoids. The maximum difference normalization method was used to normalize the metabolite content data. Each metabolite was represented by a row, whereas each sample was represented by a column. Each metabolite’s abundance was indicated by a bar of a specific color. The metabolites that were high and low were represented by different shades of red and green, respectively. The color of the bar changes from green to red as the abundance value increases.

The flavonoids and carotenoids in the grains of six Poaceae crops were investigated based on the UPLC-ESI-MS/MS and corresponding databases. A total of 201 flavonoid metabolites and 29 carotenoid metabolites were identified. 201 flavonoids including 97 flavones, 28 flavonols, 19 flavanones, 11 anthocyanins, 10 polyphenols, 7 isoflavones, 4 proanthocyanidins and 25 other flavonoids, and 29 carotenoids containing 3 carotenes, 11 xanthophylls and 15 carotenoid esters were identified (Figure 1 and Supplementary Table S1). 

In this study, we identified carotenes, xanthophylls, and carotenoid esters in all the samples, but we found that there were rare carotenes and luteins detected in rice and wheat grains. Whereas foxtail millet and maize contained more carotenoids. Rubixanthin palmitate, β-cryptoxanthin palmitate, zeaxanthin, and lutein are abundant in foxtail millet, compared with other crops. Many carotenoid metabolites, such as β-cryptoxanthin, astaxanthin, capsanthin, and capsorubin were the specific compounds in maize. In addition, violaxanthin-myristate-palmitate was only detected in broomcorn millet.

In the heatmap (Figure 1), the contents of flavonoids, carotenoids, and identified metabolites among the same crops were basically consistent and clearly distinguished from other species. Except for maize, the two cultivars didn’t categorize into the same class. Differential metabolites were predominantly engaged in flavone, flavonol, isoflavonoid, and anthocyanin biosynthesis, according to the clustering analysis of differential metabolites. The relative contents of metabolites in rice were similar to wheat, while broomcorn millet was similar to foxtail millet as well as maize. Rice and wheat contained similar flavonoids, such as kaempferol, apigenin, chrysoeriol, diosmin, and narirutin and their derivatives. Broomcorn millet and foxtail millet contained similar compounds among all kinds of flavonoids detected, and luteolin, tricin, naringenin, hesperetin, kaempferol, apigenin, chrysoeriol, and isorhamneti and their derivatives made up most of the total flavonoids. Whereas the contents of flavonoid metabolites in sorghum contrasted with those in the others greatly. As the heatmap showed that most contents of flavonoids in sorghum were significantly higher than those of other species, and there was also a great difference between sorghum species, especially 17J1344 contained more specific metabolites, such as phloridzin, delphinidin, procyanidin B3, procyanidin A3, and cyanidin derivatives.

### 3.2. PCA Based on the Differences of Flavonoids and Carotenoids

**Figure 2:**
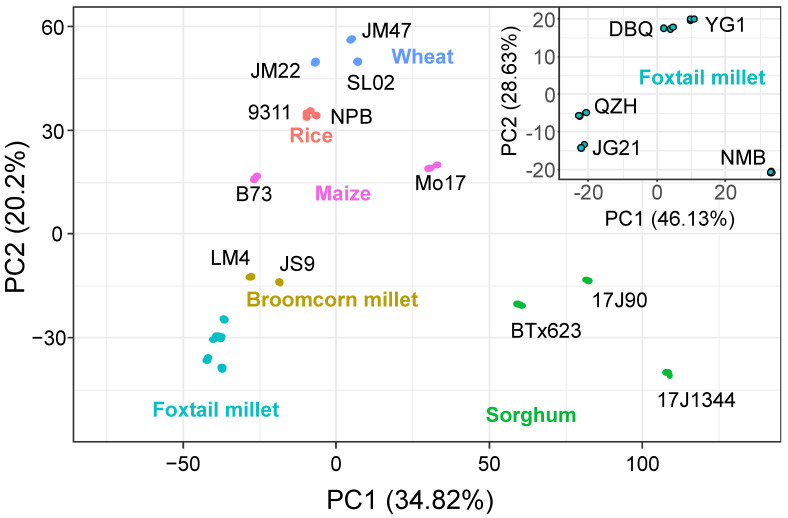
The PCA plot of flavonoids and carotenoids in all samples.

Two principal components, PC1 and PC2, represented a 34.82% and 20.2% contribution to the differences among species, respectively, and separated the six species distinctly (Figure 2). Moreover, PC1 and PC2 also revealed the differences among varieties of each species with good repeatability. The six Poaceae crops varieties were separated clearly. In addition, the staple crops (including Rice, wheat, and maize) got similar scores and were significantly separated from the minor crops (including sorghum, foxtail millet, and broomcorn millet). For instance, the 9311 and NPB rice showed slight differences on either PC1 or PC2 dimension, whereas the B73 and Mo17 maize, LM4 and JS9 broomcorn millet were distinguished mainly by PC1. Three varieties of wheat or sorghum displayed diversity on both dimensions. Five varieties of foxtail millet with disparities in edible quality were analyzed in the inner graph. The contribution rates of PC1 and PC2 explained 46.13% and 28.63%, respectively. JG21 and QZH with high eating-quality were similar on the PC1 dimension and so did DBQ and YG1 on PC2 dimension. As expected, the unpalatable cultivar NMB was far away from the four varieties.

### 3.3. Differential Metabolite Analysis

**Figure 3:**
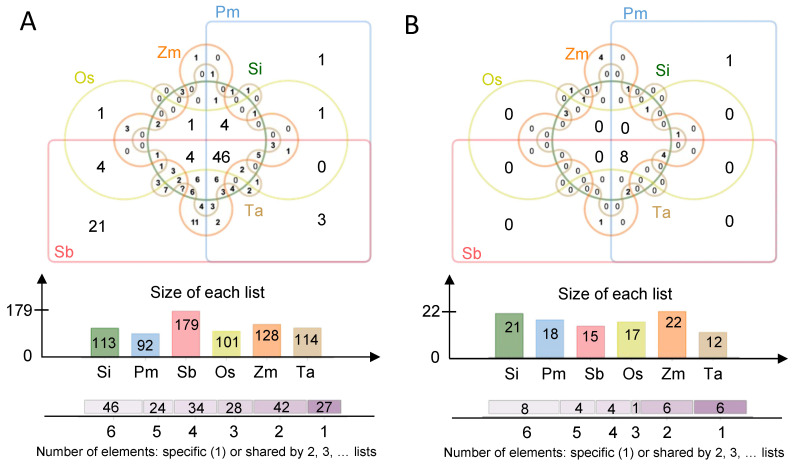
Venn diagram showing the number of flavonoids (**A**) and carotenoids (**B**) in the six species.

In this study, 114, 128, 101, 179, 113 and 92 flavonoids were identified in wheat (Ta), maize (Zm), rice (Os), sorghum (Sb), foxtail millet (Si), and broomcorn millet (Pm), respectively (Figure 3A). The annotation of flavonoids metabolite shows at supplementary Table S3. Some of them existed in grains of all the six Poaceae crops with considerable content (>19 ug/g), such as pme2977 (trihydroxyethyl rutin), pmb3894 (di-O-methylquercetin), pmc1990 (4′-hydroxy-5,7-dimethoxyflavanone) and pmf0458 (6-gingerol). However, there were also species-specific compounds. For instance, three flavonoids including pma0779 (Tricin O-rhamnosyl-O-malonylhexoside), pma6371 (di-C,C-hexosyl-luteolin) and pmf0614 (Peonidin 3,5-diglucoside chloride) were detected only in Si. Sb harbored 21 flavonoids which were not identified in other crops tested. Only one specific flavonoid for each species was determined in Os (pmb0678, 8-C-hexosyl-apigenin O-feruloylhexoside), Pm (pmb0620, Chrysoeriol 6-C-hexoside 8-C-hexoside-O-hexoside) and Zm (pmb0732, Tricin 5-O-feruloylhexoside) and no specific one existed in Ta (Figure 3A). Additionally, several carotenoids were identified in Ta (15), Zm (18), Os (21), Sb (17), Si (22) and Pm (12) (Figure 3B). The record of carotenoid metabolites is shown in supplementary Table S4. As expected, there are also the species-specific carotenoids. vio(C14:0/C16:0, violaxanthin-myristate-palmitate) and lut(C16:0/C16:0, lutein dipalmitate) were only found in Pm and Ta, respectively. Interestingly, the minor crops did not produce specific carotenoids. However, these results showed that coarse grains (Si, Pm, Sb) probably contained more specific flavonoids than staple crops (Os, Zm, Ta), whereas staple crops showed more specific carotenoids than coarse grains. 

### 3.4. Enrichment Analysis, Functional Annotation, and Differential Metabolite Screening

**Figure 4:**
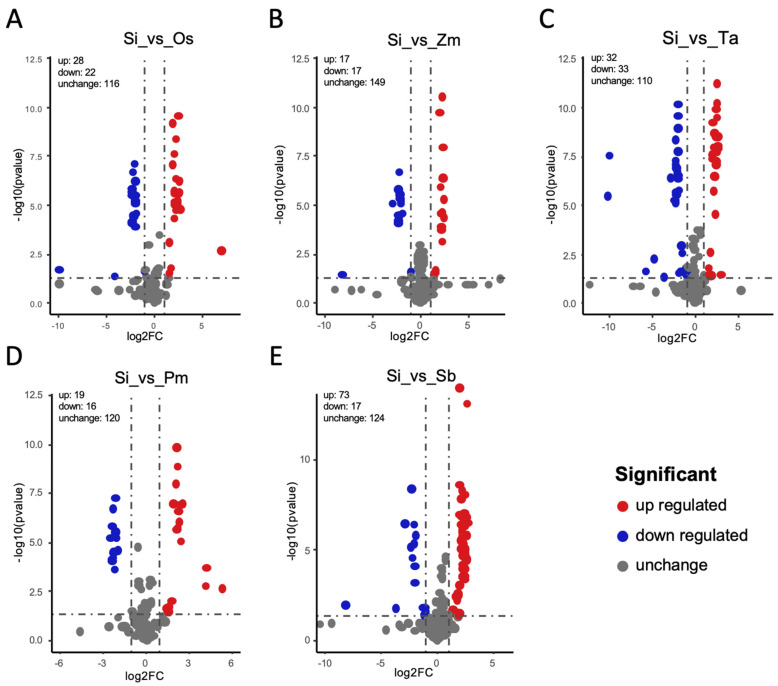
Identification of differential metabolites for five comparison groups (**A**) Si vs. Os, (**B**) Si vs. Zm, (**C**) Si vs. Ta, (**D**) Si vs. Pm, (**E**) Si vs. Sb. Differential metabolites plotted as a volcano. A metabolite is represented by each point on the graph. Up-regulated metabolites are represented by red dots, down-regulated metabolites are represented by blue dots, and metabolites with insignificant differences are represented by black dots.

**Figure 5:**
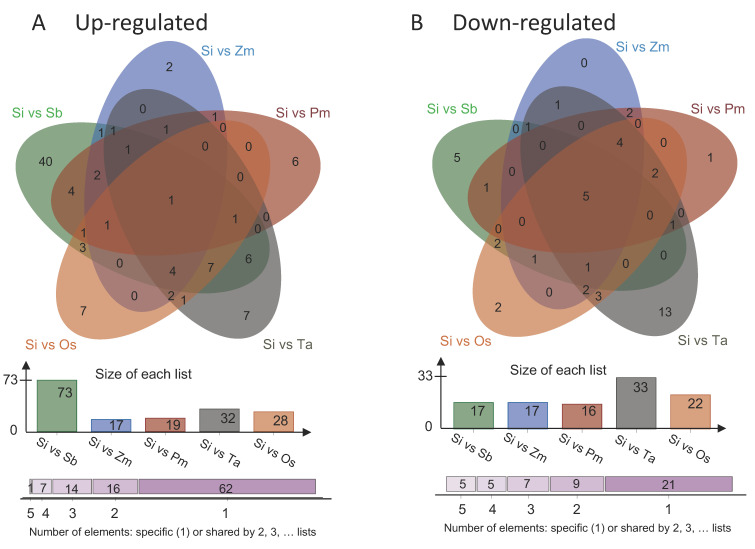
Venn diagram showing the overlapping and accession-specific differential metabolites. (**A**) Up-regulated; (**B**) Down-regulated.

By combining the variable importance in project (VIP) values of the OPLS-DA model and the fold change (FC), different flavonoids and carotenoid metabolites were screened for each comparison group. The criteria for screening included the FC ≥2 or ≤0.5 and the VIP ≥ 1.0. The screening results were presented as volcano plots (Figure 4A–E and Figure S1). We are much more interested in the differences between foxtail millet and other species, focusing on the interpretation of differences in Si vs Os, Si vs Zm, Si vs Ta, Si vs Pm, and Si vs Sb (Figure 4A–E). Identification of differential metabolites for other species comparison groups is shown in Figure S1. There were 50 metabolites that were significantly different between foxtail millet and rice (28 up-regulated, 22 down-regulated), 35 between foxtail millet and broomcorn millet (19 up-regulated, 16 down-regulated), 90 between foxtail millet and sorghum (73 up-regulated,17 down-regulated), 65 between foxtail millet and wheat (32 up-regulated, 33 down-regulated), and 34 between foxtail millet and maize (17 up-regulated, 17 down-regulated) (Supplementary Table S2). Compared with foxtail millet, most of the flavonoid metabolites of sorghum were up-regulated. Six common differential metabolites were discovered among comparison groups after intersecting each comparison group in a Venn diagram (1 up-regulated, 5 down-regulated) (Figure 5). The flavonoids caused the difference between Si and Sb, Zm, Pm, Ta, Os. Foxtail millet had a significantly higher flavonoids content, pmb0645 (6-C-hexosyl-hesperetin O-hexoside) and pma0760 (Selgin O-malonylhexoside) were the more abundant metabolites, and pma6371 (di-C,C-hexosyl-luteolin), pmf0614 (Peonidin 3,5-diglucoside chloride) and pma0779 (tricin O-rhamnosyl-O-malonylhexoside) were specific metabolites, as compared with other five crops. When compared to other Poaceae crops grains, most flavone metabolites were down-regulated in foxtail millet grains; however, flavone C-glycosides metabolites were generally up-regulated in foxtail millet. This result supported the PCA’s conclusion that flavone C-glycosides made a significant contribution to foxtail millet grains.

## 4. Discussion

Plant metabolites are essential for plant development and nourishment, and there is a great deal of metabolic variability among species [32]. Crops are indispensable for humans because they provide a variety of nutrients. Although several crops had metabolome studies, metabolic distinctions between crops have yet to be discovered. 

In this paper, we have identified a total of 230 flavonoids and carotenoids from Poaceae crops grains (including rice, maize, wheat, sorghum, foxtail millet, and broomcorn millet), which are important components of the daily human diet. Crops had a wide range of metabolic characteristics. We can focus on the differences to make more a sensible dietary organization to benefit human health, especially to increase the accumulation of nutrients and health benefits in grains and develop functional foods [16]. From the comparison of the content differences, we found that foxtail millet had natural advantages, with rich and unique nutritional value. All the luteolin derivatives, which showed strong DPPH scavenging activity [33], were up-regulated in the foxtail millet grains compared with other Poaceae crops grains. Tricin possesses anti-inflammatory and anti-cancer properties, making it a promising functional agent for glycemic control. Tricin and its compounds were found in all six crops seeds, especially foxtail millet containing more tricin 7-O-hexoside in this investigation. From the perspective of functional food, foxtail millet has a unique flavor and taste, which can be used as a supplement to people’s daily diet.

These metabolites varied widely in different crops. Foxtail millet, broomcorn millet and sorghum grains had their own unique metabolites and more abundant metabolites. The ability to increase flavonoids and carotenoids content in crops of high agronomic relevance such as foxtail millet furnishes unique opportunities to improve nutrition. For instance, rutin (quercetin 3-O-rutinoside) is a citrus flavonoid with several possible health benefits, including the strengthening of blood vessels, aiding vitamin C absorption, lowering cholesterol, preventing blood clots, and lowering blood pressure [34,35]. Rutin was found in all of the samples and was shown to be the highest among flavonol metabolites, but the variations between the samples were insignificant. The peonidin 3, 5-diglucoside chloride, belonging to the anthocyanins, which has a biomedical effect on cardiovascular diseases [36], was only detected in foxtail millet. Compared with foxtail millet and sorghum grains, most of the flavonol in grains of Poaceae crops were down-regulated. Syringetin, an O-methylated flavonol, induces human osteoblast differentiation and can possibly treat cancer [37,38]. In current research, the syringetin content in the grains of other Poaceae crops was down-regulated compared with that foxtail millet. At the same time, the syringetin derivative, that are syringetin 5-O-hexoside and syringetin 7-O-hexoside, were only detected in foxtail millet, sorghum, and maize. Kaempferide, which was solely found in sorghum and maize grains in this study, had a hypolipidemic effect on hyperlipidemia caused by high-fat meals [39]. Acacetin has been shown to have significant cardioprotective properties [40]. Acacetin also has antimutagenic, antiplasmodial, antiperoxodant, anti-inflammatory, and anticancer properties. [41]. Acacetin was found in sorghum, maize, and wheat in this investigation.

In recent years, more and more consumers have realized that diet based mainly on a single staple cereal crop is not healthy. In cereal grains, flavonoids and carotenoids are essential phytochemicals that are not only responsible for the endosperm’s distinctive color, but also confer aesthetic quality and nutritional to cereal-based products. Our results showed that flavonoids and carotenoids were species-specific, suggesting that there might be specific flavonoid and carotenoid signaling pathways in different Poaceae crops. We also found that the contents and types of flavonoids compounds in minor grains were more than those in staple crops, by comparing the differences between minor grains and staple crops. Whereas the types and contents of carotenoids in staple crops, especially maize Mo17, were more than those in minor grains, those in rice and wheat were not as high as those in staple crops. Furthermore, there were significant differences of those metabolites between different cultivars of sorghum, maize, and foxtail millet, 17J1344 (sorghum), Mo17 (maize), QZH and NMB (foxtail millet) had higher metabolite contents than other cultivars. In the future, it would be important to identify the functional genes related to these the special metabolites and ultimately improve the nutritional quality by enriching the desired metabolites in the crops by molecular breeding. Crop cultivars (rice, wheat) will have great potential to increase carotenoid content in this area, and it is also possible to consider whether homologous genes in other species to specific genes in maize can be found; then, by breeding them to increase their abundance, make them more nutritious. The ability to raise the content of flavonoids and carotenoids in crops with high agronomic value, such as foxtail millet and broomcorn millet provides unique opportunity to increase nutrition. Hence, in the future, the foxtail millet and broomcorn millet grains have considerable potential for development and utilization. Although foxtail millet and broomcorn millet have similar substance types, there were 19 up-regulated significantly different metabolites between foxtail millet and broomcorn millet, and broomcorn millet also has its own specificities, such as hesperetin, astilbin, and violaxanthin-myristate-palmitate. The two different genotypes perhaps also lead to their differences in nutrition; this requires more experiments to verify. These unique metabolites enriched the diversity of metabolites in crops while also meeting the nutritional needs of humans.

## 5. Conclusions

In this study, a total of 201 flavonoid and 29 carotenoid metabolites were detected in the grains of six Poaceae crops. Flavone, anthocyanins, flavanone, and polyphenol were the major differential metabolites, which showed species specificity. Maize, sorghum, and foxtail millet grains were rich in flavonoids and carotenoids compared with other species. The flavonoids content of the grains from 17J1344 (sorghum), QZH and NMB (foxtail millet) and carotenoids from Mo17 (maize) were higher than the other samples. These results provide insights into the accumulation patterns of flavonoids and carotenoids among different species and provide theoretical support for the application and functional development of related substances among species in the future. 

## Figures and Tables

**Table 1:** The characteristics of plant materials in this study.

Species	Cultivars	Characteristics
Wheat	JM47	Jinmai47, commercial variety
	JM22	Jimai22, medium gluten
	SL02	strong gluten
Maize	B73	female line, reference genome available
	Mo17	male line, reference genome available
Rice	NPB	japonica cultivar, reference genome available
	93-11	indica cultivar
Sorghum	BTx623	reference genome available
	17J90	edible
	17J1344	brewing
Foxtail millet	JG21	Jingu21, cultivar of high-quality conventional, reference genome available
	QZH	Qinzhouhuang, cultivar of typical representative
	YG1	Yugu1, cultivar, reference genome available
	NMB	Niumaobai, landrace
	DBQ	Daobaqi, landrace
Broomcorn millet	LM4	Longmi4, reference genome available
	JS9	Jinsu9, waxy grains cultivar

## Data Availability

All data and materials are available on request.

## Supplementary Materials

The following supporting information can be downloaded at: https://www.mdpi.com/article/10.3390/foods11142068/s1, Figure S1: Identification of differential metabolites for other species comparison groups; Table S1: A list of the 230 flavonoids and carotenoids metabolites detected in the in the six species; Table S2: A list of the differential flavonoids and carotenoids metabolites between foxtail millet and other species; Table S3: The annotation of flavonoids metabolite; Table S4: The annotation of carotenoids metabolite.
