# Supplementary figures and images for: Differential Flavonoids and Carotenoids Profiles in Grains of Six Poaceae Crops
Source: Foods. 2022 Jul 12;11(14):2068. doi: 10.3390/foods11142068 (PMC9325323; doi:10.3390/foods11142068)

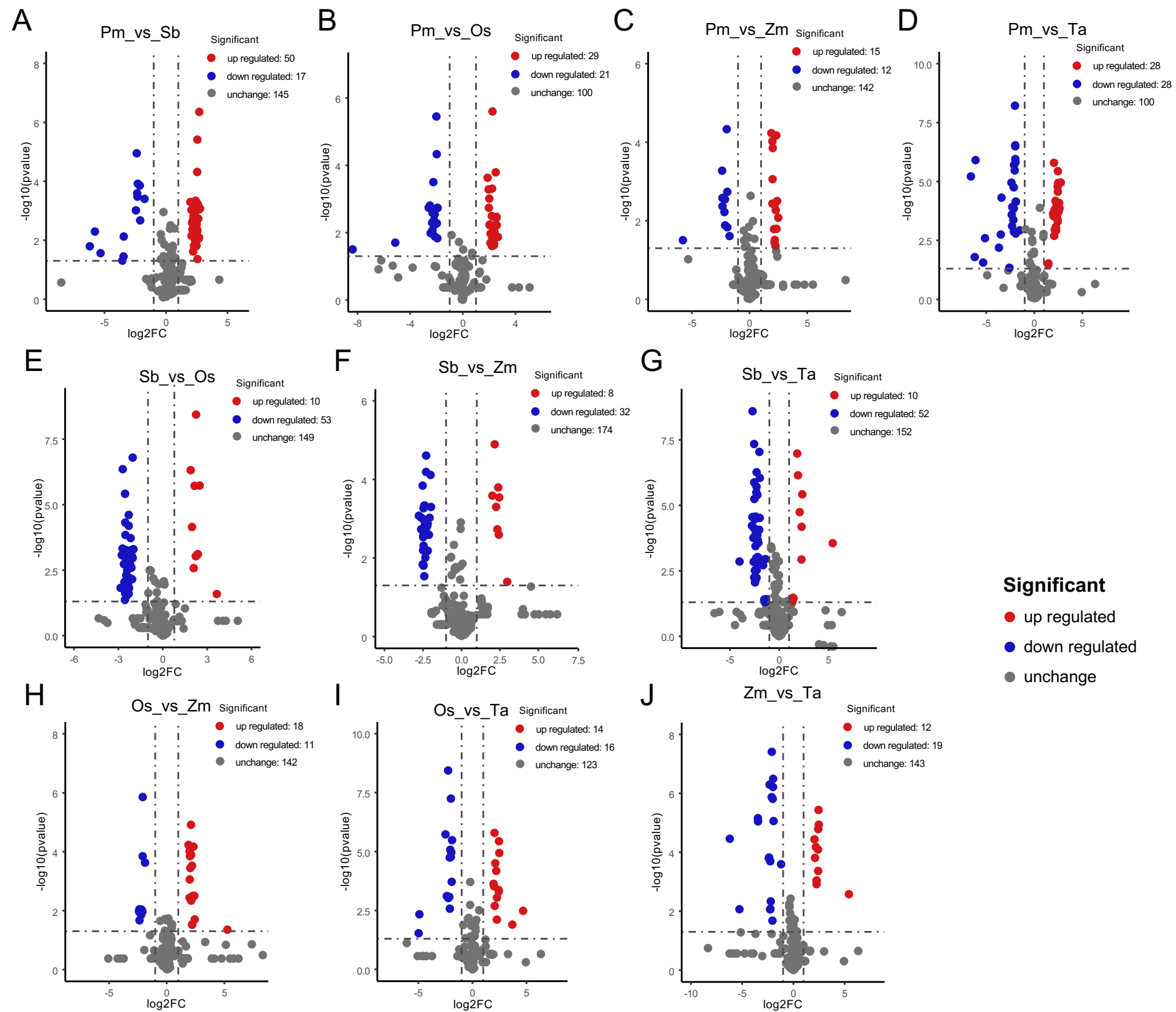

Supplement: Supplementary file 1 [file foods-11-02068-s001.zip › Supplementary Figure S1.pdf]
